# Recent Advances in PLA Stereocomplexes: Synthesis, Modification, and Applications

**DOI:** 10.3390/polym18161969

**Published:** 2026-08-13

**Authors:** Haowei Cui, Yottha Srithep, John Morris

**Affiliations:** Manufacturing and Materials Research Unit, Faculty of Engineering, Mahasarakham University, Mahasarakham 40000, Thailand; 67010363001@msu.ac.th (H.C.); j.morris.and@gmail.com (J.M.)

**Keywords:** PLA, stereocomplex, biodegradability, biomedical use, micelles, agricultural

## Abstract

Stereocomplexes derived from natural polylactides are an important class of renewable plastics. Although neat polylactides generally exhibit low elongation at break and low Young’s modulus, stereocomplex formation significantly enhances their thermal resistance and stiffness (rather than toughness), dimensional stability, and controlled degradability, compared with simple polylactides. Consequently there has been significant research efforts to discover improved uses of these stereocomplexes—formed when the two enantiomers are combined—reported in many previous reviews. Here, we discuss improvements in them reported in the last five years, including a wide range of applications from simple packaging to biomedical aids, which make use of polylactide biodegradability, and self-assembing micelles, with quite complex structures, that are used for drug delivery and agricultural uses. We also noted that many general claims for degradability in PLA based polymers need to be qualified: the polymers will degrade, but very slowly in normal, low humidity conditions. However, degradation may be accelerated by heat, moisture, and enzymes—and also by fabricating with functional groups.

## 1. Introduction

Plastics formed from lactides, the derivatives of lactic acid, are found in natural—and thus renewable—sources, as distinct from those formed from finite petroleum sources. These building blocks are chiral, i.e., they have two forms—the naturally occuring L-lactides and their mirror images, D-lactides-which react identically, if the reactants are non-chiral, but only chemically synthesized. Both can be polymerized, but these polymers lack many desirable properties of polymers from petroleum sources. However, their natural sources allow them to degrade slowly in the environment and thus potentially reduce the pollution that petroleum-based polymers cause by cluttering and damaging it. Although degradation in normal environments is slow, it can be accelerated by heat, moisture, introduced functional groups and enzymes (e.g., in composting). Thus, considerable research effort has focused on improving the lactide-based polymers so that they can replace the finite resources from petroleum-based plastics that we now rely upon in almost all aspects of our lives—from packaging to tools.

**Figure 1:**
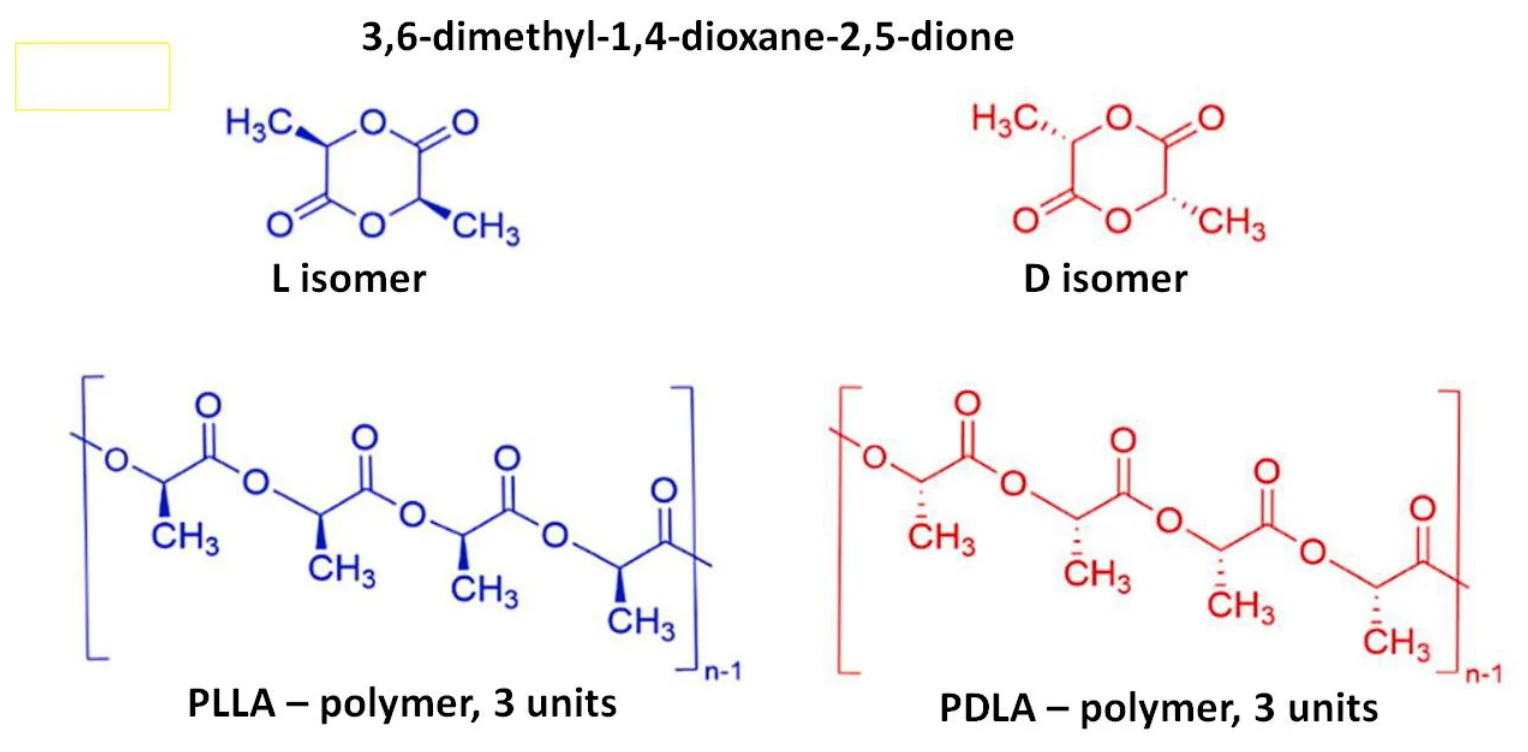
L- and D-lactide monomers and PLLA or PDLA polymers—usually formed by ring-opening polymerization of the lactide monomers.

**Figure 2:**
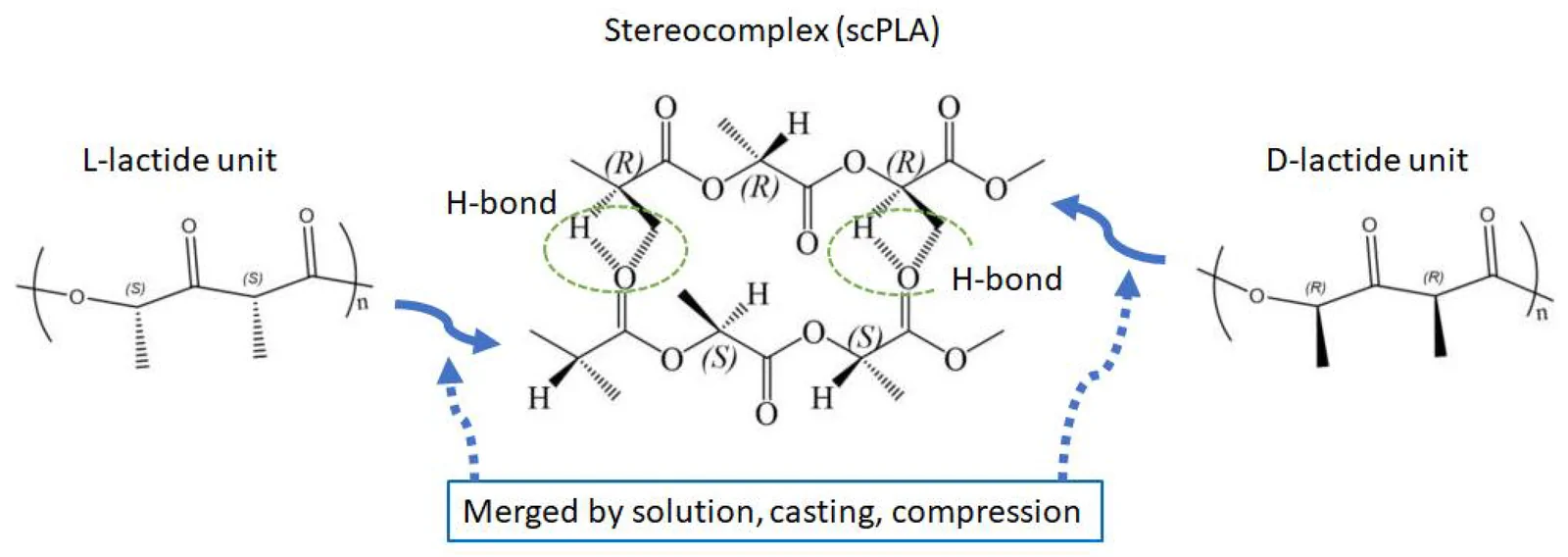
Hydrogen bonds linking L- and D-enantiomers; left and right—L- and D-enantiomers; center—stereocomplex showing H-bonds linking the two.

Mixtures of L- and D-lactides will, in the right conditions, form stereocomplexes, in which the L- and D-monomers (shown in Figure 1) are weakly bound—as shown in Figure 2. The discovery of the distinct properties of stereocrystals formed by polymerization of stereocomplexes has sparked new efforts to take advantage of the new properties of these polymers, e.g., better toughness, flexibility, and degradability. This has led to several reviews covering their properties (mechanical, thermal, permeability, optical, etc.) and applications (packaging, medical, etc.)—listed in Table 1. These reviews are extensive and readers are referred to them for the history of these valuable crystals: this review focuses only on new developments in this area in the last few years—from 2021 to mid 2026.

### 1.1. Contributions

Since these reviews cover many details of syntheses, properties, and applications of work before 2021, we provide only brief details and here focus on new reports in the previous five years.

### 1.2. Overview

Section 2 describes developments in the formation of the stereocomplexes themselves: it focuses on the physical formation of the crystals—controlling the phases, gelation, crystallization, etc., and techniques to prefer stereocrystals rather than homocrystals. New steps in processing and manufacturing of polymers containing the stereocomplexes are reviewed in Section 3. New proposals to enhance properties, incorporating blends and composites, are set out in Section 4. Enhanced properties are described in Section 5. Newly proposed applications of developed products, including biomedical uses—including self-organizing micelles—are set out in Section 6. Finally, we summarize key developments in Section 7 and conclude in Section 8.

## 2. Strategies to Enhance Stereocomplex Formation

### 2.1. ‘Pure’ Stereocomplexes

Both ‘pure’ enantiometric monomers can be polymerized—using several methods—to form pure polymers, which will, under appropriate conditions, crystallize to form ‘homo-crystals’, containing only one of the enantiomers, e.g., PLLA or PDLA—see Figure 1. The relative availability and lower cost of the more available PLLA leads to more work focusing on PLLA. However, since Ikada et al. [16] discovered that mixtures of the two enantiometric lactides would form ‘stereocrystals’ (scPLA), which exhibited several desirable properties (e.g., tougher, dramatically higher (∼50 °C) melting points, and less permeable), a strong focus has been on the potential benefits of mixtures and scPLAs have been studied extensively to take advantage of these improvements. PDLA is generally produced by ring-opening polymerization (ROP) of enantiometrically pure D-lactide, which is the common industrial route. Earlier laboratory studies also reported the separation of D-lactide from mixtures of enantiomers using optically active resolving agents or chromatographic methods; however, these approaches are costly and impractical for large-scale production.

An early extensive review by Tsuji et al. [15] covers most early work and has been followed by general reviews by Wi et al. [1], Michell et al. [17], and Raef et al. [5] and texts by Mulchandani et al. [18], Chapter 9 of Ray and Banerjee’s book [6], and Auras et al.’s 2nd edition [10].

Much recent work has attempted to increase the average stereocomplex molecular weight and crystallinity, partly challenged by the lower availability and higher cost of the D-isomers than the readily available and naturally produced L-isomers. Many have attempted to compensate for this by creating grafts with other degradable polymers—discussed in later sections on grafts and multiple block polymers (Section 4.1.1). An interesting strategy, particularly for medical applications, synthesized structures of several polymers, or micelles, which are effective for carrying drugs to target sites, is discussed in Section 6.4.7.

Typical synthesis paths produce mixtures of scPLA crystallites and homocrystallites; these mixtures, containing scPLA, are generally amorphous and show fewer benefits, so some have also tried to increase the fraction of scPLA crystallites to take full advantage of their enhanced properties [1]. Also, many researchers applied multiple improvement techniques, including blending with other polymers, fibers, or nanocomposites so there are similarly many cross-references in this review.

### 2.2. Phase Changes

Noting that the scPLA melting point is typically 50 °C higher than that of pure PLLA or PDLA, controlling the liquid to solid change has been used as one technique to control the scPLA fraction. Several recent works have further discussed this in detail with regard to crystal growth—see Section 2.3.8—and miscibility—see Section 2.3.9.

#### 2.2.1. scPLA Exclusive

Higher fractions of scPLA crystals could take advantage of their enhanced properties and the conditions (e.g., temperature, relative amounts of PLLA and PDLA, etc.) which prefer homo- or stereo-crystal formation have been extensively discussed. A 2020 review by Luo et al. discussed techniques for preferring scPLA crystal formation and thus these enhanced properties [12]. Further, Liu et al. were able to achieve exclusive scPLA crystallization from the glassy state in dilute solutions by sufficiently fast spin-coating, whereas a slowly evaporated solvent allowed phases to separate and form predominantly homocrystals: they used the Flory–Huggins theory to explain that phase separation was avoided by rapid solvent evaporation [19]. Yao et al. also reported that thermal control of melt mixes was important to efficiently nucleate the mixes and return high stereocrystal yields [20]. The order in which the various PLA conformers could appear was discussed by Zhang et al.: at 3000 K/s, they observed $10_{3}$ helices first, but, after the chains were able to interact, $3_{1}$ helices appeared [21]. Similarly, Huang et al. observed that fast cooling, allowing the enantiomeric chains to move, favored the formation of stereocrystals [22].

Zheng et al. noted that the activation energy for complex formation was reduced, altering the crystal structure—see Section 2.3.8 [23]. J. Zhu et al. also reported that they could convert homocrystals by mixing, then spinning in a critical temperature range—187 °C–197 °C—at which they achieved 60% conversion to scPLA [24]. In another method to increase the scPLA fraction, Zha et al. used Zn acetate (Zn(OAc)_2_) to strengthen intermolecular interactions and form scPLA crystals alone: they needed only 0.01 wt% to achieve this [25].

#### 2.2.2. Stereo Random Copolymers

Generally, scPLA polymers are formed from chains of L- or D-lactide units permitting partially matching alignments of L- and D-units, but Tsuji et al. reported the effect of lengths of sequences of monomer units. They also noted that small (∼3%) amounts of D-monomers allowed PLLA chains to pack more efficiently in the α crystals [26]. Additionally, alternating chiral centres-with L-D or D-L and poly(L,D-lactic acid-alt-l-2-hydroxybutanoic acid)-P(DLA-alt-L-2HB) or P(DLA-alt-D-2HB) were synthesized and evaluated: Tsuji et al. proposed that these novel combinations allowed preparations with a variety of mechanical and degradability characteristics [27]. In one variation of this concept, Yuan et al. built double-grafted polymers, with both PLLA and PLDA side chains, and used them to enhance scPLA crystal yield and avoid homocrystallization: they needed only 0.5 wt% to avoid any homocrystals [28]. Zheng et al. also discussed using various tacticities to vary crystallization and resultant properties [29].

Several have also increased scPLA content; for example, in Section 4.3, Zhang et al. stabilized PLLA/PDLA with cellulose, in a Pickering emulsion, leading to only scPLA with high melt stability so that high heat resistance was maintained over multiple processing steps [21].

### 2.3. Fabrication Methods

Due to the strong potential for scPLA to improve performance, many fabrication methods have been reported (see Table 2). The discussion following focuses on new techniques;

#### 2.3.1. Ring Opening Polymerization and Chain Extenders

Ring-opening polymerization remains the primary route for synthesizing well-defined PLLA and PDLA precursors with controlled molecular weight, narrow molecular weight distribution, and tailored chain architectures. However, following significant progress in single active site catalyst systems for stereoselective ring-opening polymerization of lactides, Sun et al. discussed the effects of metal complexes on the stereoselective polymerization of lactides, including metal center types and ligand structures [45]. These catalysts, capable of regulating polymerization and stereochemical control, influence the subsequent formation of scPLA crystallites. Sun et al. reviewed the effects of metal-complex catalysts and highlighted the important roles of both metal centers and ligand structures in determining polymerization efficiency and stereoselectivity [45]. Improved catalyst design has enabled preparation of higher-molecular-weight PLLA and PDLA chains with enhanced potential for stereocomplex formation, while also facilitating the synthesis of block, graft, and other advanced architectures for biomedical and functional applications.

In an scPLA blend with TPS, Srithep et al. used epoxy groups in Joncryl ADR4370 to extend the chains, reduce the melting enthalpy, and generally improve mechanical properties [32,33].

#### 2.3.2. Compatibilizers

Compatibilizers are crucial to allow incompatible polymers to form high molecular weight polymers: in one new example, Yilmaz et al. [39] discussed the use of polyurethane, polyhedral oligomeric silsesquioxane (POSS), amic acid isobutyl (AAI), tetra silanol phenyl POSS (TSP), and Joncryl (JO) in electrospinning of fibers. AAI led to the smoothest ‘super hydrophobic’ surface (contact angle 154 °C) and 812 nm diameter. JO led to the strongest (315 MPa) fibers, highest crystallinity (20.1%), and highest temperature durability (292.16 °C) ([39]). Li et al. showed that epoxidized soybean oil as as a reactive compatibilizer allowed preferential formation of scPLA crystals [46].

#### 2.3.3. High Molecular Weights

Achieving high molecular weight scPLA crystals still remains a challenge, addressed by several studies which include using scPLA particles as nucleating agents in homopolymers, overcoming limits on molecular weights for scPLA blends ([4]), enhanced crystallization (see Section 2.3.4 [28]), and formation of multiple block polymers (see Section 4.1.1).

#### 2.3.4. Crystallization

As with crystal formation in almost any material, crystallization is encouraged by nucleation—the addition of small seeds on which crystals grow—but in stereocrystals, a possible unique feature is that the ‘nuclei’ may form at the interfaces as the two enantiomers are blended together and scPLA crystals form (see Section 2.3.7).

#### 2.3.5. Nucleation

Purnama et al. tested both PDLA and scPLA as nucleating agents for PLA homopolymers: they found that scPLA enhanced mechanical properties of PLLA up to 5% scPLA content, but after that it affected brittleness. Although PDLA could induce stereocomplex formation, preformed scPLA crystallites exhibited a stronger nucleating effect [47].

Similarly, Baimark et al. [48] used low molecular weight scPLA to nucleate and form high molecular weight PLLA with complete scPLA crystallites, prepared by melt blending. The LMW-PLLA powder homogeneously melted in the high molecular weight PLLA matrices with good phase compatibility. Enthalpies of crystallization ($\Delta H_{c}$) on cooling HMW-PLLA increased and the half crystallization time ($t_{\frac{1}{2}}$) dramatically decreased as the scPLA powder content increased [48]. Trofin et al. [49] also observed ‘self-nucleating’ behavior of scPLA crystals in melts: PLLA crystallinity doubled with scPLA addition, but a six-fold increase was noted for PDLA. Different morphologies were also associated with the crystallinity—lamellar spherulites or flow-induced fibrillar ‘shish-kebabs’. They also confirmed increased heat deflection temperature with increased scPLA content [49]. Miscibility between PLA and PVAc-modified cellulose nanocrystals (CNC) enhanced chain motility and suppressed homocrystallization, encouraging scPLA formation. Nucleation by the PVAc-CNC also reinforced the mechanical and thermal properties of the generated nanocomposites [50].

Nucleation naturally affects crystal growth and crystal molecular weight, by both small particles (Section 5.3), polymer chains themselves, and micelles (Section 6.4.7).

Korber et al. investigated the effect of nucleating agents on crystallization, including NA-21 (aluminium hydroxy bis[2,2′-methylen-bis(4,6-di-tert-butylphenyl)phosphate] (molecular weight: 1015.22) in combination with talc, at up to 70 K/min cooling [51].

#### 2.3.6. Gelation

*e*-form crystals found in PLLA/PDLA gels were found to transiently change to the α-form at ∼75 °C before forming stereo complexes in gels: Praveena et al. noted that understanding this phase change could be used to explain re-organization into the helical polymers [52].

Crystallization (particularly scPLA crystals) was shown to be enhanced by small amounts (0.5%) of double grafted co-polymers, which led to preferred scPLA crystals ([28]): Yuan et al.’s double grafts, formed by a “grafting-condensation” with oligosiloxane backbones followed by hydrolysis, then condensation, encouraged high molecular weight blends containing sc-crystals. Longer lignin-PLDA blocks were more effective to nucleate stereocrystals in PLLA-lignin-PDLA blends: these blends were also stronger and absorbed UV-light more strongly [53].

Wang et al. used a variety of norbornene dicarboxylate complexes (TMX-Na, Mg, Al, or Ca) as stereo-selective nucleating agents: TMX-Al led to only scPLA, with 50% crystallinity, excellent melt memory, and VST up to 145 °C [54].

Full stereocomplexation was achieved by Yao et al. using finely dispersed scPLA particles in a melt-mixing procedure, which mixed PLLA with 0.75 wt% PDLA at elevated temperatures to homogeneously mix PLLA/PDLA chains, followed by a low temperature, permitting full stereocomplexation [20].

AlOOH nanorods were used by Rong et al. to simultaneously ensure compatibility and nucleate scPLA crystals, leading to rapid crystallization and improved thermal resistance [55].

#### 2.3.7. Interfacial Crystals

Interfaces of all types can be nucleation sites. scPLA crystals were observed at the interface with carbon nanofibers [56] and graphene platelets [57]: in both cases, improved performances were noted compared to structures based on PLLA alone. Interfacial scPLA crystals were also formed by Li et al. (2025) after pre-reacting PLDA with a compatibilizer: this formed a structure which stabilized the PBAT particles in their blend [58]. Koike et al. compressed sheets of PLLA and PDLA to form scPLA between them and affect the hydrolysis rate and maximum tress before failure—multiple sheets increased strength, but smaller numbers of sheets increased hydrolysis rates [59].

#### 2.3.8. Crystal Growth

**Figure 3:**
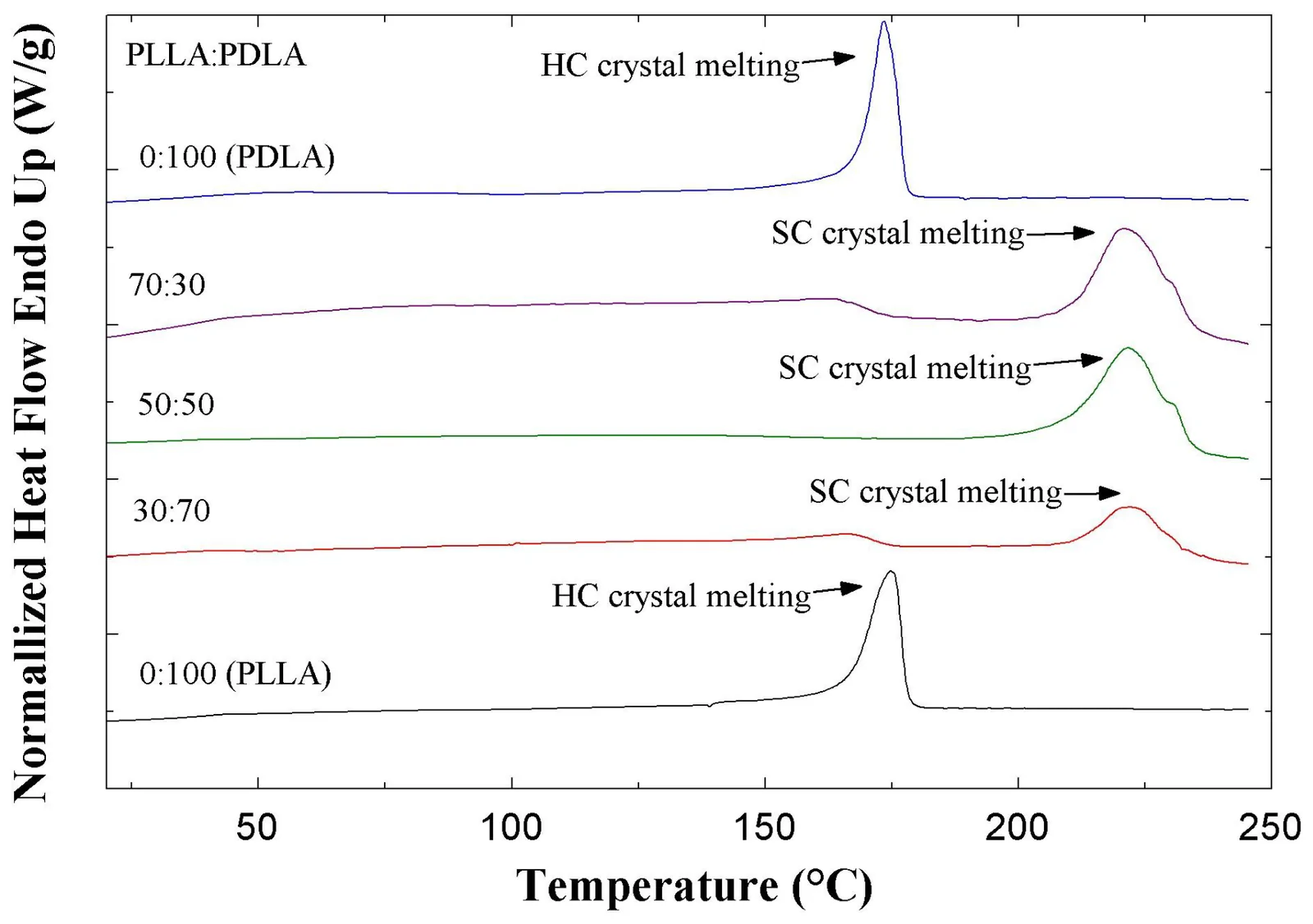
DSC thermograms of PLLA/PDLA blends with different blend ratios illustrating homocrystal (HC) and stereocomplex crystal (SC) melting behavior. Pure PLLA and PDLA (HC) melt at ∼175 °C, whereas PLLA/PDLA blends (SC) melt in the range 220–230 °C, confirming stereocomplex formation.

The conditions (e.g., temperature, relative amounts of PLLA and PDLA, etc.) which prefer homo- or stereo-crystal formation have been extensively discussed. Yao et al. noted that thermal control from melt mixes was important to efficiently nucleate the mixes and return high scPLA yields [20]. The DSC thermograms in Figure 3 show how far the melting points differ, with scPLA crystals (SC) melting at much higher temperatures than the pure (HC) crystals. The order in which the various PLA conformers could appear was discussed by Zhang et al. —at 3000 K/s, they observed $10_{3}$ helices first, but after the chains were able to interact, $3_{1}$ helices appeared [21]. Chain movement in fast cooling, which favored the formation of stereocrystal formation, was also noted by Huang et al. [22].

To avoid the higher cost of PDLA in scPLA, a low cost fibrillation step, which produced stretched 5–10 µm fibers, aligned the chains and improved crystallization and subsequent tensile performance in fibers, even with low (<3 wt%) PDLA content [35]. Sun C. et al. also noted that reducing chain entanglement led to higher scPLA crystal proportions [60]. Similarly, Sun M. et al. reported that, with a high temperature heat setting in their polyester fiber production line, they obtained almost complete scPLA structure at 190 °C and better hydrolysis resistance in their drawn fibers [61]. Zheng et al. noted that the reduced scPLA formation activation energy led to regularly folded stereocrystal lamellae [23]. Zhu et al. also reported that they could convert homocrystals by mixing then spinning in a critical temperature range—187 °C–197 °C—at which they achieved 60% conversion to scPLA [24].

As expected, Lv et al. showed that adding scPLAs with higher molecular weights crystallized at higher temperatures [62], but they also observed the Mpemba effect [63], which they attributed to chain motion and non-equilibrium conditions approaching the crystallization point. Zhou et al. observed that when the two enantiomers had exactly the same chain length, they crystallized together into uniform stereocomplexes with extended chain conformation; they also discussed how the excess segments of the longer component remained amorphous or complexed with other short chains [64].

Effects of melt-annealing time on scPLA formation showed that H-bonding between PMMA and grafted cellulose nanocrystals relaxed over time so that controlling scPLA crystallization must be understood to design new PLA materials [65].

Cui et al. studied the effect of the L:D ratio and observed the morphologies, including, for example, the serrated edges in 50:50 blends vs. smooth lamellae in the pure enantiomeric version [66].

Higher relative amounts of scPLA were observed as the blend temperature approached the 220 °C scPLA melting temperature. Increase in PDLA content had the same effect: the crystallization rate also increased with scPLA content. They also observed a Vicat softening and temperature rise from 67.8 °C to 164.7 °C in a 1:25 PLLA:PDLA blend, enhancing heat resistance and also resistance to hydrolytic decomposition [67].

Cao et al. showed that adding PDLA and controlling the temperature below the homocrystal melting points improved barrier performance for packaging and food storage (see also Section 5.6): although homocrystals were formed, they were removed during crystallization at 190 °C [68,69]. Similarly, Ahmadi et al. [70] reported that the growth of the stereocomplex crystals vs. homocrystals was affected by temperature: their Hiller-type models showed how to influence the development of different stereo complex crystal structures (either nodular or coarse lamellar structures) via mesophses growth [70].

##### Crystal Growth Front

Chen et al. attributed variations in nucleation efficiency to concentrations of $10_{3}$ helices close to the crystal growth front: they suggested that understanding the mechanisms promoting $10_{3}$ and $3_{1}$ helix formation should guide design of new scPLA based materials [71].

##### Stereocrystals

As shown in Figure 2, L- and D-enantiomers are able to neatly link together in long chains with H-bonds joining chains of opposite chirality.

L- and D-chains may be joined by multiple techniques (see Table 3). In crystals, these chains arrange themselves into stacks of twisted triclinic $3_{1}$ cells, containing monomers linked by H-bonds (see Figure 2-center.)

#### 2.3.9. Miscibility

Immiscibility between polymer components has been widely reported as causing difficulties in forming novel combinations. Interestingly, Chen et al. [72] reported that high molecular weight PLLA and PDLA had differing miscibilities: their random polymer of PL(D)LA with caprolactone showed that PLLA was completely miscible, whereas PDLA was immiscible. Their AFM data showed well-defined interfaces in PLLA or PDLA blends, confirming phase separation. They noted that this had significant implications in understanding stereocomplexes [72] and generally required the use of suitable compatibilizers (see Section 2.3.2).

#### 2.3.10. Strain Induced

Stretching-induced chain alignment promoted scPLA crystal nucleation—reported by Tuccitto et al. [35] and Liao et al., who also noted evident strain induced crystallization of PLLA/PDLA in injection moulded samples; they attributed this fast creation of order to large numbers of scPLA particles forming crystallization nuclei. They compared this with reorganization caused by heating [34].

#### 2.3.11. Tuning of scPLA Content

Developments in controlling crystallization to produce high-molecular-weight scPLA content were reviewed earlier in Section 2.2.1 [23] and Section 2.3.8, where the Mpemba effect was also reported [62].

Layered double hydroxide fillers were used by Chen et al. to suppress homocrystal crystallization increasing scPLA context: they claimed economic benefits for these hydroxides as well as lower degradations caused by excessive filler content [73].

#### 2.3.12. Enantiomer Detection

**Figure 4:**
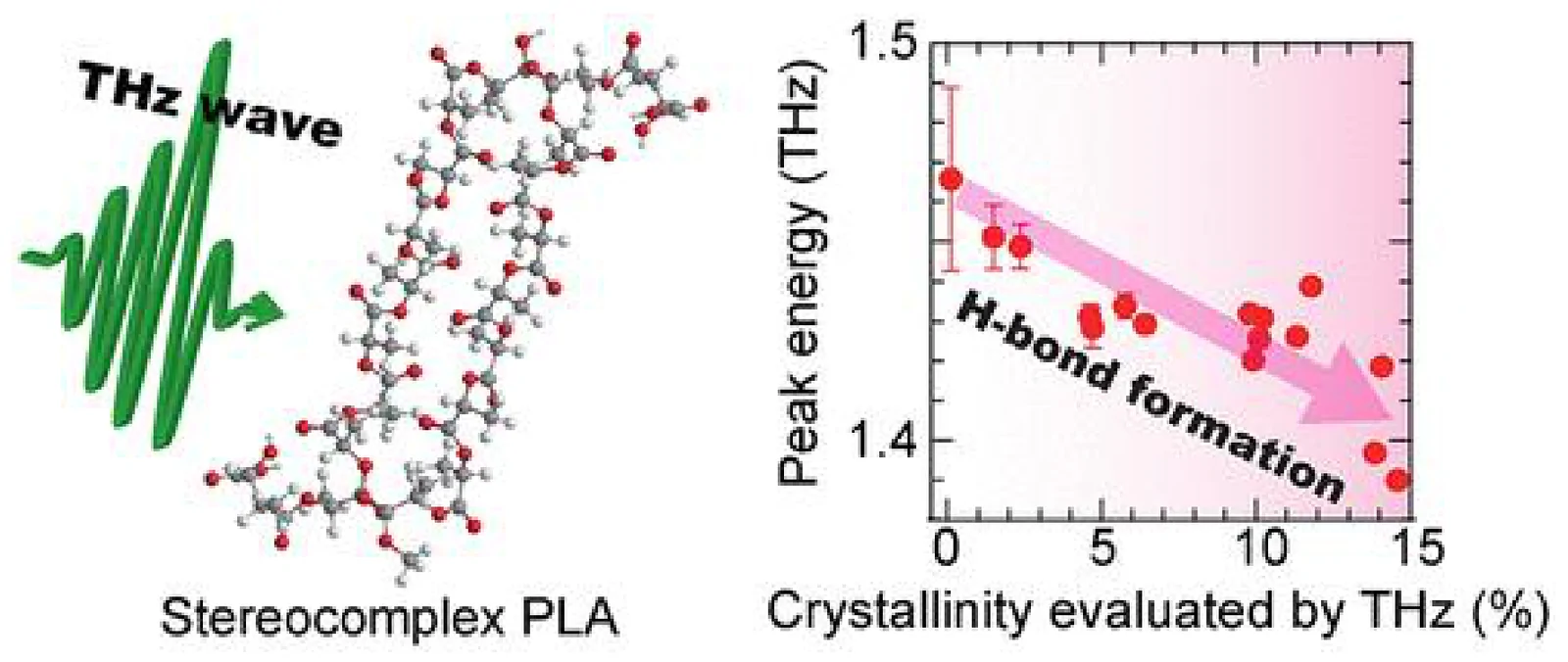
**Left**: scPLA crystal: center L- and D-lactice chains positioned to allow H-bonds linking L- and D-chains. **Right**: crystallinity derived from H-bond detection—copied with permission from Hirata et al. [75] © American Chemical Society.

The potential damage of large amounts of the not-natural D-lactides in humans makes the measurement of relative amounts of the L-, D- and LD-mixtures important to diagnose the rare medical problems attributed to D-lactides: although Pohanka’s review [74] concluded that D-lactides were not highly toxic and were not serious threats to humans, his review reported many observed instances of problems induced by D-lactide excesses, thus clinicians need to be aware of this possibility and test for it. Previous methods have required HPLC, GC-MS, enzyme assays, or electrochemical analysis and present difficulties in sensitivity, cost, and selectivity. However, recently terahertz spectroscopy has been used to rapidly and accurately distinguish between the chiral components [75,76]. In particular, Hirata et al.’s THz spectroscopy showed the positioning of the H-bonds between the enantiomers (see Figure 4). Additionally, Li et al. designed a dual-resonant THz detector, capable of discriminating 1 ng/mL of PLA enantiomers [76].

## 3. Processing and Manufacturing

New variations on most processing and manufacturing steps continue to be reported, including screw extrusion, melt spinning, solution casting, and fiber drawing: a summary listing advantages and noted limitations is in Table 3.

**Table 3:** Strategies: advantages vs. limitations.

Strategy	Mechanism	Advantages	Limitations	Studies
Molecular	↑ chain diffusion	↑ simple, scalable	Limited for high MW	[64,77]
weight control	and scPLA yield			
Chain	↑ chain length and	↑ chain entanglement,	↓ reactive processing	[33]
extenders	interchain interactions	mech performance		
Compatibilizers	↑ PLLA/PDLA	Enhance scPLA	Biodegradability ↓?	[39,46]
	mixing	formation		
Nucleating	↑ heterogeneous	Faster crystallization	Additive dependence	[47,48,49]
agents	nucleation			
Annealing	Larger crystals	High crystallinity	↓ processing time	[20,60,61]
Nanofillers	Nucleation and	Multiple property	Dispersion challenges	[55,77,78]
	reinforcement	improvements		
Block or graft	Molecular design	Tailored capability	Complex synthesis	[28,79,80]
copolymers	approach			
Star polymers	↑ scPLA	↑ scPLA stability,	↓ complex synthesis,	[41,81]
	interactions	thermal resistance	↓ production cost	

Notes: ? indicates further research needed. Nanofillers include CNC, CNF, graphene, lignin, etc.

### 3.1. Screw Extrusion

A continuous screw extrusion process was able to produce 95% conversion to scPLA crystals with the correct conditions (temperature controlled up to 240 °C) [40].

Supmak et al. demonstrated that, after extruded films were cooled and then exposed to microwaves, the chains rearranged and formed stereocomplexes (see Section 5.2 [41]).

### 3.2. Melt Spinning

Marx et al. achieved 57% crystallinity in a scPLA mix vs. 47% for a melt-spun mix that was strong enough for ‘textile strengths’ (50 cN/tex) [82].

### 3.3. Bicomponent Fibers

In melt-spinning, Huang et al. generated scPLA fibers in situ: they noted that phase separation was inhibited in the fibers, leading to exclusive scPLA formation and they claimed that this process was a facile and effective way to pure heat resistant scPLA crystals [37]. Variations in the domain structures due to formation of scPLA at the PLLA-PLDA interface in melt-spun fibers was reported by Zhao et al. [83]. Other scPLA uses in fibers are noted in Section 2.2.1, Section 2.3.2, Section 2.3.8 and Section 6.2. Heat resistance was also added by carbon nanofibers that were well dispersed among scPLA crystals formed in situ (see Section 5.2).

## 4. Blends and Nanocomposites

Blends with PLA and other polymers or nanoparticles have continued in the search for better biogradable plastics (see Table 4). Also, the following Section 6.4.7 (“Micelles”) discusses developments in complex self-forming structures, which are being exploited mainly for biomedical applications.

### 4.1. Structural Modifications

Varied PLA structures have been built by chemically bonding other polymers to form grafted or multiple block polymers. Blends with nanocomposites and graphene derivatives have also been reported (see Section 4.2).

#### 4.1.1. Grafted and Block Polymers

Many grafted or ‘block’ polymers which combined scPLAs and nanocomposites to form ‘stereo-nano’ materials were reviewed by Samsuri et al. (2023): they noted their potential to generate new materials [4].

After studying asymmertic diblock copolymers with Monte Carlo simulations, Xu et al. concluded that the fraction of ‘beads’ of different blocks was the key factor controlling scPLA formation: it could be adjusted though different bead contents of the different blocks [79].

Zhu et al. compared stereo-complexes derived from several block polymers and found that the cyclic block polymers were more miscible, leading to improved formation of the scPLA polymers [80]. Double-grafted copolymers with both PLLA and PDLA side chains were shown to efficiently promote crystallization and thus yield of scPLA crystals in high-molecular weight blends: Yuan et al. believed that the unique copolymer was crucial to suppress phase separation of the opposite enantiomers during melting, i.e., acting as an efficient compatibilizer, encouraging the generation of alternatingly arranged PLLA/PDLA chain clusters, thus favoring scPLA nucleation and crystal growth [28].

Correlations between PLLA:PDLA ratios and crystallinity of PCL block polymers were shown to allow fine tuning of overall properties by Mandlule et al. [84].

Choi et al. also formed rotaxane molecules, using the α-cyclodextrin in the PEG part of pseudo-polyrotaxane, and studied several wheel-axles to provide some insights into these complex host–guest interactions [85,86]. They also studied various combinations of L- and D-polymers with PEG copolymers to form scPLA structures: their samples added urethane caps [87].

#### 4.1.2. Tertiary and Higher Order Blends

The potential of blends between PLA and PBS, constrained by their inherent incompatibility, has already been reported. Blends with hydrophilic PEG were shown to affect gelation and allow more compact packing [88]. Xu et al. [89] have added more suggestions to overcome this; they showed that an epoxy-based copolymer (ESA) encouraged ring-opening with the terminal carboxyls of both PLA and PBS and toughened the blends as well as increasing thermal stability, hydrophobicity, and optical clarity. Epoxy groups in ESA also increased degradation. Thus, higher order blends could be ‘engineered’ to adjust desirable properties. As an example, Zhu et al. reported core and shell assemblies using ternary blends of PLA, PGA, and PBS blends: they concluded that engineering the interfaces using these blends led to improvements in brittleness and better barriers for $O_{2}$, extending shelf life [90].

#### 4.1.3. Four Arm and Star Polymers

Star shaped block copolymers from scPLA and PEG were exploited to quickly crystallize scPLA: the PEG segments increased chain mobility and backbone flexibility [91]. PLA-PEG copolymers, with PEG sheaths and a star of four PLA ‘arms’ linked to hepta methylene linkers, were built with opposite chirality blocks by Signori et al. With hepta methylene linkers, they exhibited temperature dependent gelation, but with amino group linkers, thermally irreversible gels were formed [81] Supmak et al. also built star polymers with high temperature resistance (see Section 5.2). Interestingly, when formed into films by extrusion and thermoforming as food packaging trays, they initially showed low crystallinity because they were cooled rapidly in initial processing. However, used in microwaves, the EM field interacted with chain polar groups, heating them to slightly higher temperature than the critical $T_{g}$ and triggering chain rearrangements to form stereocomplexes, enhancing both heat resistance and shape stability [41]. Similarly, scPLA was generated ‘in use’ by Buchatip et al. [92].

‘Bottlebrush’ polymers are another group of multiple-armed polymers that allow functions to be tuned by varying compositions of the ‘arms’: their potential was reviewed by Li et al. [93].

#### 4.1.4. Nanocomposites

In their review, Samsuri et al. showed that scPLA nuclei as well as nanocomposites improved physical (e.g., electrical, thermal, mechanical, and melt stability) properties, as well as biomedical ones [94].

#### 4.1.5. Amphiphillic Blocks and Micelles

Amphiphillic blocks, which can automatically assemble into micelles are commonly used for drug delivery, are discussed in more detail in Section 6.4.7.

#### 4.1.6. Surface Modification

One specific modification style altered the surfaces, primarily of films: surface modified films were prepared with PDLA and low molecular weight poly(d-lactic acid-co-glucose) copolymer (PDLAG) by melt polycondensation. These films had scPLA crystals on the surface and homogeneous crystals (HC) in the bulk. With increasing immersion, PLLA films modified by PDLA decreased both HC and SC structure, whereas PLLA films modified by PDLAG increased scPLA structure and decreased HC structure. Hydrophilic glucose surface PDLAG residues improved the hydrophilic nature of surface-modified PLLA films. ScPLA crystals improved heat resistance of modified PLLA films, but glucose residues blocked crystallization and promoted thermal degradation. The improvement in thermal stability, hydrophilicity, and crystallization is essential in order to obtain useful PLA-based biomaterials [95].

After modifying both ends of scPLA chains with different end groups, Asano et al. found their modified stereocomplexes kept the expected thermal degradation temperature improvements (see Section 5.4 [96]).

#### 4.1.7. Other Polymer Blends

The potential of blends between PLA and other naturally degradable polymers, e.g., PBS, TPS, PMMA, and others, is constrained by their inherent incompatibility, thus agents to make them more compatible are usually required: Table 4 lists examples and also notes when the stereocomplexes where formed in the interface between phases.

**Table 4:** Blends with other polymers.

Polymer	Compatibilizer	Solvent	Notes	Ref
		or Melt		
TPS	Glycerin	M	Plasticizer	[32,33]
	Joncryl ADR-4370		as chain extender	
PBS	PDLA/PBS	M	i-SC; toughness; heat	[30]
		S	PBS as plasticizer; ↑ tensile strength	[97]
	ESO/ECD	M	Ternary PLA/PBS/PGA-Core/shell blends	[90]
	ESO	M	Preferential scPLA	[46]
	GNP	S	Thermal conductivity via graphene platelets	[36]
	ESA	M	↑ thermal stability, hydrophobicity, optical clarity	[89]
PMMA	CH_3_Cl	S	Films; ↑ elongation, ↑ thermal stability	[98]
PGA	ESO/ECD	M	Ternary PLA/PBS/PGA	[90]
PBAT	i-SC	M	scPLA for in situ nucleating	[99]
		M	↑ tensile strength, modulus, thermal stability;	[100]
			PBAT ↑ ductility	
	SG	M	Co polystyrene-co-glycidyl methacrylate effect	[58]
	PMMA	M	3D printing	[42]
PCL		M	ϵ-caprolactone, shape memory	[101]
PEG	S	G	Sol-gel transition	[88]
		M	Differing chain ends	[87]
	cat	G	4-arm neutral or cationic linkers	[81]
		S	PEG-based Star polymers	[91]
	Buf	G	Delivery of deferoxamine	[102]
PHEEAA		M*	Crystallization self-assembly	[103]
2HB		M	Alternating chiral centres	[27]
lignin		M	PLDA blocks were more effective to nucleate	[53]
			stereocrystals in PLLA-lignin-PDL	
CA	Bn	S	Changing chain ends	[96]
PC	BPM	M	Rubbery interface compatibilizer	[104]
PPC	GO	M	poly(propylidene carbonate)	[105]
HA	i-SC	M	Hydroxy apatite bone scaffolds	[106]
Graphene	i-SC	S	Thermal properties via graphene platelets	[36]

Note: i-SC = interfacial stereo complex; cat = cationic; S = solvent;M= melt; G = gel; M* = micelle; Buf = phosphate buffer; chain = chain extender; SG = polystyrene-co-glycidyl methacrylate.

### 4.2. Graphene Blends

Graphene blends typically targeted high temperature resistance or conductivity in specific applications. Hydroxylated graphene nanoplatelets had PLLA and PLDA grafted onto them through ring opening polymerization and then were melt compounded. The resultant composites—compared to pure PLLA—showed higher thermal conductivity (by 390%—$1.1\;{\mathrm{Wm}}^{-1}K^{-1}$) and a 65 °C higher heat distortion temperature of 200 °C [57].

Liu et al. (2021) improved poly(propylidine carbonate) blends by adding scPLA crystals at the interface to link graphene oxide in their blends [105]. High molecular weight blends were also achieved by blending small amounts of graphite oxide; Zhong et al. observed that scPLA formed gradually and only scPLA was formed when 2 wt% graphite oxide was added: they attributed this to interactions between PLA chains and graphite oxide surface hydroxyl groups [77].

Fullerene was also used as a nucleator to improve the scPLA crystallization and form high molecular weight crystals: Chang et al. reported that 1 wt% fullerene was sufficient to prevent homo-crystallization [107].

### 4.3. Cellulose Blends

Fang et al. showed that grafts with cellulose nanocrystals and PLLA and PDLA combinations improved the interfacial tensile modulus and strength and noted that PLLA/cellulose nanocrystal grafts crystallized almost twice as fast as equivalent PDLA crstals. Tyhus, there were significantly different crystallization kinetics [108]. In their Pickering emulsion approach, Zhang et al. also stabilized PLLA/PDLA blends with regenerated cellulose, forming cellulose/scPLA microspheres [21].

### 4.4. Other Stereocomplexes

Stereocomplexes can be formed from polyesters based on α- or β-hydroxy acids: they were reviewed by Bandelli et al. [14] and Tutoni and Becker [109]. Similarly, Popowski et al. observed stereocomplexes from two aliphatic polyesters—(poly((R)-epoxide-alt-(R)-anhydride) and poly((R)-epoxide-alt-(S)-anhydride))—that changed to semi-crystalline polymers with only one stereo-center inversion and showed a melting temperature $T_{m}=238$ °C or ∼20 °C higher than their constituents [110]. Polymandelic acid stereocomplexes were also reported by Tsuji et al. [111] and Lee [112]. Remarkably, Tsuji et al. reported that these polymandelic acid stereocomplexes showed melting temperatures (105 °C–127 °C) that were much lower than homocrystallites (162 °C–180 °C): similarly, thermal degradation energies for the enantiomers were lower than for other enantiomers [27,111]. Additionally, Duti et al. reported stereocomplexes in peptide gels [113].

### 4.5. Adding Nanofibers

Fillers, silk fibroin nanodisks, were blended with scPLA to enhance scPLA crystallization and suppress homocrystals—not only was the crystallization half-time decreased, but the scPLA fraction increased [114]. Ren et al. used cellulose nanofibers and claimed that 3% nanofibers led to complete scPLA crystals with no homocrystals: they attributed this to interchain interactions between the nanofibers and PLA [78]. Cellulose fibers were also used by Fang et al. [108], Ren et al. [78], and Zhang et al. [21] (see Section 4.3).

## 5. Properties

New polymers, ‘engineered’ to target specific properties, are mentioned in this section.

### 5.1. Toughness

Polylactides notably break at low stress and thus are unsuitable for many purposes: although stereocomplex formation improves strength and thermal resistance, PLA remains brittle unless additional toughening strategies are introduced. PBS was used by Srithep et al. [97] and Yang et al. [115]; PBAT by Veang-In et al. [100] and other polymers listed in Table 4.

Another example grafted lignin nanoparticles into PLLA using PCL as the compatibilizer: further, PDLA partially replaced some PLLA to increase the notched impact strength from $2.3\;\mathrm{kJ}\;m^{-2}$ to $15\;\mathrm{kJ}\;m^{-2}$ [115].

### 5.2. High Temperature Resistance

The ability to safely heat packaged food in microwaves is clearly important in today’s ‘fast-food’ environment: Supmak et al. showed that adding 0.1–0.2 mole fractions of star PDLA to PLLA blends and heating to slightly above the $T_{g}$ led to chain re-arrangements favoring scPLA crystal formation and higher heat tolerance whilst maintaining stable shapes [41]. Pasteurization requires both heat resistance and shape stability: Buchatip et al. developed star-shaped polymers with a PCL macroinitiator with 20% added PDLA, improving the properties compared to PLLA. In these applications, heating during use led to more scPLA being formed [41,92].

Gu et al. saw the formation of scPLA crystals using PLLA and PBS as compatibilizers and nucleation sites, leading to heat resistances of $E_{80\;{}^{\circ}C}>680\;\mathrm{MPa}$ [36].

Carbon nanofibers were prepared and well dispersed among stereocomplex crystallites formed in situ, leading to extremely high heat resistance and thermal conductivity ($2.3\;{\mathrm{Wm}}^{-1}K^{-1}$, 1000% greater than that of the PLLA) [56]. Similarly, good compatibility between nanosilica and scPLA ensured no air bubbles were formed and the particles distributed evenly in the matrix. Crystallinity increased—24% with 10 wt% of nanosilica— and decomposition temperature was ∼33 °C higher than neat scPLA [31].

Norbornene dicarboxylate complexes were used as stereo-selective nuclear agents to facilitate exclusive scPLA forms with over 50% crystallinity, leading to a dense network, increasing the high heat resistance, with a Vicat softening temperature >=∼145 °C [54].

Nanocomposites containing PLA-PCL-lignin nanoparticles were also used to enhance temperature resistance [115] (see also Section 5.1).

Yan et al. blended melts between the stereo crystal and homocrystal melting points, producing powders with varying crystallinity. Injection molding within the scPLA melting range preserved most of the preformed stereocrystals, leaving materials with crystallinity of ∼42.1% and a Vicat softening temperature of 136.6 °C—notably with with no additives or further processing. Further, it was strong and heat tolerant and suitable for industrial use and production using conventional equipment [116].

Introduction of nanosilica into a melt-blended PLLA/PLDA mix allowed the nanosilica particles to be evenly dispersed and encouraged scPLA formation—the degree of crystallinity rose from 17% to 24.3% as the nanosilica content increased from 0 to 10%. Thermal decomposition was also 33 °C higher as well as tensile strength and modulus [31].

### 5.3. Thermal Conductivity

To improve thermal conductivity, Li et al. added Al_2_O_3_ to PLLA/PDLA blends and observed scPLA crystals formed with Al_2_O_3_ at 3 to 40 vol% and retained on cooling. At 40 vol%, a thermal conductivity of $1\;{\mathrm{Wm}}^{-1}K^{-1}$ was obtained [117]. In another technique, using graphene nanoplatelets, scPLA granules were melt blended with 1:1 PLLA:PDLA, then coated on the surfaces from solution blending, finally segregated, and double-percolated structures were formed into PLLA/GNP@SC composites through compression molding, leading to $0.56\;{\mathrm{Wm}}^{-1}K^{-1}$ conductivity [36].

A carbonate based network restricted scPLA chain mobility and led to a 106.5 °C heat deflection temperature as well as an impact strength of $7.5\;\mathrm{kJ}\;m^{-2}$ [104].

### 5.4. Thermal Degradation

Degradation temperatures of enantiomeric polymandelic acids were also recorded to be much higher than scPLAs ([111]). Similarly, degradation temperatures of scPLA melt-blown nonwovens, measured by Yu et al., increased with the incorporation of scPLA crystals: the maximum rate of decomposition increased to 385.5 °C: degradation by proteinase K was also reduced [118].

In a series of modifications of both ends of scPLA chains, in experiments using diacetyl caffeic and cinnamic acids as end groups, Asano et al. showed that modified stereocomplexes could maintain their predicted modifications, e.g., thermal degradation temperature improvements [96].

#### Fire Retardancy

Aluminium diethyl phosphinate (ADP) plus PDLA improved fire retardation: at 15% ADP in a PLLA/PDLA/ADP blend, the crystallization temperature rose to 175 °C and heat distortion temperature rose to 170.4 °C, with no loss in mechanical properties [119].

### 5.5. Hydrolytic Degradation

Degradation was measured in a Langmuir film balance to measure the effect of molecular weight by Gavande et al. [120]. Measurements of degradation in fine melt-blown mats of fibers showed that amorphous and crystalline portions of homocrystalline PLA fibers in the mats were subject to both surface and bulk erosion by hydrolysis but the scPLA rich regions were only surface eroded: the higher surface area of these fine mats led to relatively fast decomposition in both water and compost. Crystal content led to better hydrolysis resistance as well as improved mechanical and thermal performance [121]. Compressing PLLA and PDLA layers together to ‘bond’ them with an scPLA interface increased tensile modulus and strength, but also degradation [59].

### 5.6. Mass Transport

Chen et al. reviewed using scPLA to enhance gas and vapour barriers [122]. They further discussed modulating the barrier by controlling the various crystal forms ($\alpha ,\alpha^{\prime }$ and scPLA) that could be used to affect barrier properties, which were correlated with crystallinity, amount of amorphous phases, and the ‘free’ volume: they noted that $\alpha^{\prime }\gg scPLA>\alpha$, with $\alpha^{\prime }$ showing the highest permeability for both oxygen and water [123]. A background to these effects was noted in Section 2.3.8 [72].

Effective oxygen and water barriers for use in packaging were fabricated by Cao et al.: they decreased oxygen permeability by 76% (from $1.59\times 10^{-14}$ to $0.38\times 10^{-14}\;{\mathrm{cm}}^{3}\cdot \mathrm{cm}/{\mathrm{cm}}^{2}\;\mathrm{sPa}$) and water vapour by 70% (from $6.78\times 10^{-14}$ to $2.04\times 10^{-14}\;{\mathrm{cm}}^{3}\cdot \mathrm{cm}/{\mathrm{cm}}^{2}\;\mathrm{sPa}$)) [68]. Further, they showed that chain lengths of 4- and 6-armed PLAs could be set by choosing the initiator in ring-opening synthesis, which in turn affected crystallization. Overall, new materials with enhanced gas barriers could be developed [69].

To evaluate film permeability, films with PLLA:PLDA compositions from 85:15 to 30:70 and homocomplex PLLA and PDLA were formed and annealed at 160 °C for 5–30 min to understand the effect on crystallinity, permeability, and functional and mechanical performance. As expected, crystallinity and permeability coefficient increased with annealing. As little as 15% PDLA acted as a nucleating agent and induced crystallization in ≤5 min. Further blended annealed films showed significantly better moisture barriers [124]. At high crystallinity, Li et al. (2024) [125] confirmed a high correlation between barrier properties at high crystallinity levels, but a low correlation at low crystallinity. Nevertheless, the scPLA crystals improved barriers [125].

## 6. Applications

### 6.1. Foams

Srisuwan et al. improved the water resistance of thermoplastic starch foams by dip-coating them with PLA-PEG copolymers blended with PDLA, where the phases were compatible with foam surfaces. Overall, the stereo complexes improved water resistance of the foams [126].

Gao et al. made use of the six hydroxyl groups of d-mannitol as nucleating agents to increase the scPLA content from 23% to 46%: the enhanced cell structure increased the storage modulus of their foam at 220 °C to 11.8 MPa g/cm^−3^ or almost 12 times higher than PLLA/PLDA alone foams [127].

### 6.2. Fibers

Zhao et al. formed scPLA in the interface in regions aligned along their melt spun fibers and noted that their fibers crystallized in a way that differed substantially from fibers formed from solutions in previous work. They also reported that annealing of the solid fibers caused crystalline or stereocomplex domains to be re-distributed [83]. In a similar process, fibers with PLLA sheaths and PDLA cores were spun from melts, but then laser-heated: the molecular orientation in the fibers increased with the spinning velocity, leading to ultra-fine fibers of highly oriented scPLA crystals [38]. Introducing shear flow while blending was shown to create a more densely packed structure (compared to a ‘pearl necklace [128]) with increased ultimate tensile strength. Sun et al. suggested this as another method to target performances [128].

#### Oil–Water Separation

Nanofiber membranes, spun from SiO_2_-PLA-cellulose diacetate composites, were able to clean oil-contaminated water: they were found to be strongly hydrophilic, yet strongly oleophobic (with contact angles of 0° for water but >160° for oil) [129] and claimed promise for handling emulsified oily water.

### 6.3. 3D Printing

To generate materials adequate for 3D printing, Yang et al. used a toughened quaternary scPLA/PBAT/PMMA system: to effectively blend scPLA with PBAT to add toughness and ductility, they controlled the compatibilization with PMMA to evenly dispersed the system components to achieve complete scPLA contents. They also noted that the non-reactive, readily available, and cheaper PMMA as the compatibilizer was more effective [42]. Lyu et al. toughened their scPLA/PBAT blend with $SiO_{2}$, evenly dispersed at up to 5 wt%. In 3D printing, they relied on the nozzle temperature to form scPLA and enhance the tensile strength [44].

### 6.4. Biomedical

de Franca et al. reviewed blends, copolymers, and composites with scPLA mostly focusing on biomedical applications [7].

#### 6.4.1. Dental

Mats formed from scPLA fibers containing up up 10% metronidazole were used by Srithep et al. to treat periodontal disease: their drug loaded spun fiber mats showed higher sustained releases of the drug for up to seven days with higher scPLA content as well as higher fiber tensile strengths. Increased scPLA content also led to lower cytotoxicity [32].

#### 6.4.2. Bone Repair

In bone repair applications, surface modification of the nano-HA/PLA composite with PDLA improved the mechanical performance by forming stereocrystals at the interface: this was attributed to improved dispersion of the PDLA in the HA/PLLA matrix [130]. Similarly, Shuai et al. grafted PDLA to hydroxyapatite (HA) scaffolds in a PLLA matrix, and then incorporated them into a PLLA matrix: in this case, interfacial scPLA complexes chemically grafted by H-bonding formed a scaffold in which the tensile strength (compared to the basic PLLA-HA scaffold) increased by 86% and modulus by 69% [106].

Park et al. showed that adding scPLA to PLA bone plates enhanced mechanical properties as well as transparency in the visible and near IR regions [131].

#### 6.4.3. Bone Engineering

Chuan et al. used nanofibers and scPLA to build membranes, compatible with bone marrow stem cells: they grafted hydroxyapatite and scPLA. Mechanical tests showed increased tensile strength (30%) and Young’s modulus (35%) vs. previous strengthened composite nanofiber membranes [132].

#### 6.4.4. Stem Cell Therapy

A hydrogel scaffold was built with a triblock polymer composed of MPEG, scPLA, and PEI for delivering α-cyclodextrin. Significant upregulation of the markers, including HOXA10, ITGB3, and E-cadherin, were observed in in vitro experiments, and confirmed in in vivo ones [133].

#### 6.4.5. Vascular Stents

Stents were 3D printed using long chain branching structures with good crystallization characteristics and mechanical strength to use as vascular stents [43].

#### 6.4.6. Drug Delivery

Im et al. reviewed uses of scPLA in three applicatons: anti-cancer therapy, tissue engineering, and defeating dangerous microbes [134].

Amphiphillic copolymers containing multiple glucose groups were synthesized with PLLA and PLDA-co-glucose by melt condensation, leading to a multi-armed amphiphilic result with unreacted OH groups on the surface. Glucose groups acted as nucleation sites and no homo-crystals were formed, improving the melting temperature by ∼50 °C. The contact angle dropped to $77.5^{\circ }$ with 5% glucose. The enhanced crystallinity, hydrophillicity, and thermal stability were claimed useful for drug delivery and tissue engineering [135].

PLLA, PDLA, and chitosan (CHI) prepared by spray-drying were loaded with the antiretroviral drug, tenofovir alafenamide, by Narayanan et al.: they confirmed their structures were safe and effective against JurkatEf cells infected with HIV [136]. They also tested the effect of aluminium oxide nanoparticles on long-acting oleogels laden with the same nanoparticles for anti-HIV therapy [137].

#### 6.4.7. Micelles

**Figure 5:**
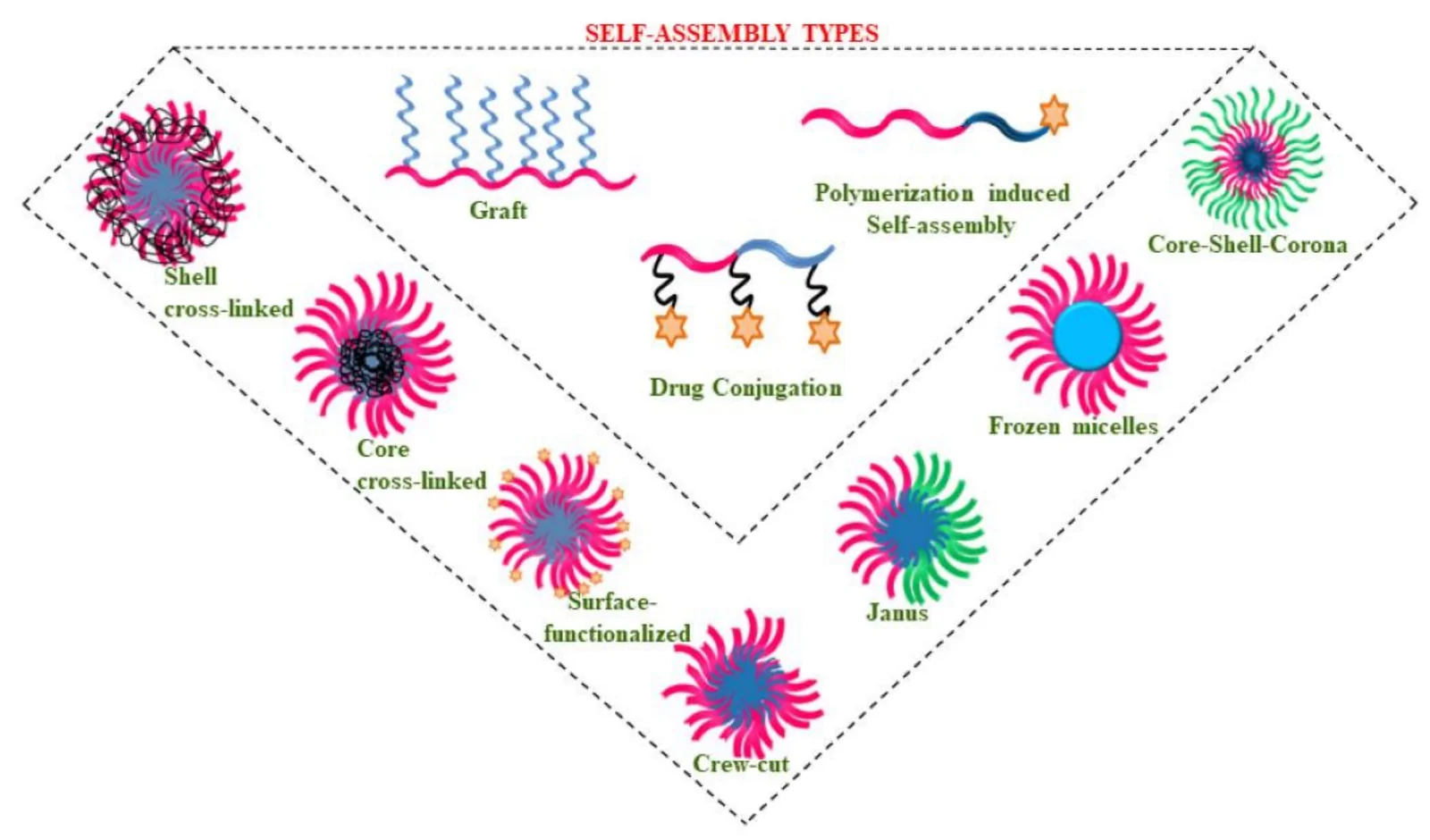
Examples of micelles structures. Taken from Kuperkar et al. [9].

In related drug delivery work, several groups have described ways to build amphiphilic block polymers that will assemble themselves into micelles, which can encapsulate drugs into their structures and deliver them effectively to target regions, e.g., cancers or tuberculosis bacteria (Figure 5).

In an early example, Kianni et al. prepared cholesterol-augmented scPLA nanomicelles to transfer rifampicin to *Mycobacterium tuberculosis* to stop it developing drug resistance: in vivo studies demonstrated significantly higher reduction in *M. tuberculosis*, so these rifampicin-loaded micelles were claimed as a stable, biocompatible, and efficient nanocarrier system with better drug uptake [138]. In another system, Munez-Lopez et al. defined a framework (using PEG-block-PLA diblock copolymers) to design PLA blocks that can be assembled into micelles [139]. Phase transitions driven by stereocrystal formation in coil-rod-coil(PLA) cylindrical nanoparticles were described by Xie et al. [103]: they used self-assembled triblock coplymers of PHEAAm-b-PL(D)LA-b-PHEEAm. They noted that the aging temperature, block composition, and the solvent was important in determining the chain exchange kinetics and believed that their results guide attempts to further optimize understanding of these systems [103]. As a basis of their structures, these block polymers and their ability to assemble themselves were discussed by Kuperkar et al. in 2022 [9]. Thermo-responsive PNIPAAm-b-PLA amphiphilic block copolymer micelles were built by Ghasemi et al. [140]. Schaller et al. built Janus (the ‘gatekeeper’ of Roman mythology, had two faces-enabling him to look in both directions at the same time) (or ‘patchy’) micelles-spherical micelles with a scPLA core and a separate ‘corona’ of polystyrene/poly(tert-butyl methacrylate) (PS/PtBMA) blocks, which were efficient compatibilizers for the immiscible PS/PtBMA blends [141].

A further type of scPLA micelles—self-assembled from PEG-block-PLLA and poly(2-(dimethylamino) ethyl methacrylate) (PDMAEMA)-block-PDLA—were used to fabricate hybrid scPLA-Au nanocarriers, where active drugs were encapsulated within the core: whereas the Au nanoparticles were held in the shell via reduction of ${\mathrm{AuCl}}_{4}^{-}$ by PDMAEMA [142].

Drugs were efficiently loaded in hypoxia-responsive scPLA micelles to inhibit breast cancer metastasis in an orthotopic murine model by Lu et al. [143]. Thermoresponsive hydrogels of PLA and PEG copolymers, which allowed targeted and controlled release of deferoxamine, a clinically applied iron-chelating drug, were built into micelles by Bikiaris et al. [102].

#### 6.4.8. Ocular Drugs

Assiri et al. have reviewed promising results from the use of micelles for delivery of ocular drugs—noting their smaller size, self-assembly, and thus easier preparation and sterilization and solubility than other nanocarriers [3].

### 6.5. Agriculture

#### Pesticide Control

Avermectins are naturally derived lactones (from *Streptomyces avermitilis*) used as broad-spectrum agricultural insecticides. They kill parasites by binding to glutamate-gated chloride channels, causing paralysis, and are considered safe, with minimal side effects, at therapeutic doses. From scPLA, Wang et al. built tunable avermectin-loaded scPLA nanospheres with a regular spherical shape and controlled-pH-responsive-release [144].

## 7. Summary

In an earlier summary (Table 3), we contrasted advantages and limitations of the scPLA fabrication strategies. with representative studies using them in the period 2021–2026. This section lists key advances reported in this period in Table 5 under the lead authors. It is notable that the variants listed there are not complete, and several authors have suggested strategies which can be ‘engineered’ to create new variants tailored to achieve target properties or applications (see methods listed in Table 3 and papers by Yuan et al. [28], Zhu et al. [90], Chen et al. [71], Im et al. [134], Cao et al. [69] and Qi et al. [135]).

## 8. Conclusions

As we show, the potential of stereocrystals formed in polylactides to overcome weaknesses in biodegradable plastics has led to an intense research focus over the years, since Ikada discovered them in 1987 [16]. Improvements in the last five years cover several areas reported in this paper. These efforts start with basically improving the yield of stereocrystals in ‘pure’ polylactides, mainly by controlling the thermal processing of melts [20] and the move to blending with other polymers [32,97,100,101] to acquire their properties without sacrificing degradability, and thus their environmental attractiveness. These blends generally involve additional agents to make the new polymers compatible with the basic polylactides: some of these ‘compatibilizers’ have used groups, e.g., epoxides, within the agents to chemically bond with the base polymers. More complex techniques have chemically ‘grafted’ new polymers to the base lactides to enhance mechanical properties and heat resistance required by new applications. Applications targeted by improvements have ranged from packaging to medical ones, e.g., stents or bone repair, and even agricultural use [144].

An exciting recent use has described self-organizing micelles, which can encapsulate critical drugs to facilitate their delivery within our bodies, e.g., to target cancer sites, as discussed in Section 6.4.7.

Needless to say, the environmental benefits of slow, but adjustable, natural degradations alone, in addition to, in particular property benefits, ensure that the potential for these sterreocrystals will cause considerable further research in areas only suggested by the recent additions set out in this paper.

### Challenges and Future Perspectives

Challenges for further use of scPLA and derivatives remaining are:

- Higher fractions of scPLA crystals vs. homocrystals
  - Partially satisfied but effects on other properties need further analysis
  - scPLA stability during fabrication
- High molecular weights to enhance mechanical properties, while
  - Maintaining higher scPLA crystal fractions
  - Keeping costs, i.e., PLDLA components, down
- Potential for other blends, nanoparticles, etc.
  - Many combinations have been assessed, but many further possibilities exist
- Exploitation of micelle structures
  - Potential for controlled drug release
  - Possibility to target specific structures, e.g., cancers, only partially explored
  - Regulatory issues for extended use in animals
- Degradability and recyclability
- Scalability or the ability to ‘scale up’ a process which is successful in a laboratory to industrial scale.

Additional note: The Mpemba effect, reported by Lv et al. [62], has the potential to affect crystallization rates, so conditions to induce it might be further studied.

## Figures and Tables

**Table 1:** Previous reviews of PLA stereocomplexes.

Year	Lead Author	Focus	Refs	Cite
2025	Wi	In situ scPLA formation	210	[1]
2025	Hu	Advances and New Materials Engineering	163	[2]
2024	Assiri	Block copolymers; ocular systems	171	[3]
2023	Samsuri	Stereocomplexation and nanocomposites	133	[4]
2023	Raef	General	201	[5]
2022	Ray	Sustainable PLA blends	bk	[6]
2022	de Franca	General, but Biomedical focus	159	[7]
2022	Lopez-Pelaez	scPLA: synthesis for biomedical applications	th	[8]
2022	Kuperkar	Amphiphilic block polymer micelles	151	[9]
2022	Auras	Poly(lactic acid): Synthesis to End of Life	bk	[10]
2021	Somnuake	Electrospun scPLA nanofibers with super-hydrophobic surfaces	th	[11]
2020	Luo	Mini-review: Stereocrystal conditions	76	[12]
2020	Zhao	Super tough PLA blends: review	269	[13]
2019	Bandelli	Stereocomplexes; polyesters other than PLA	205	[14]
2016	Tsuji	Stereocomplexes-10 yrs report	310	[15]
1987	Ikada	Stereocrystals-first report	16	[16]

Notes: General = review covering many aspects; bk = book; ch = chapter; th = thesis.

**Table 2:** Variations on stereocomplex fabrication techniques (2021–2026).

Method	References
Melt blending	[30]
+injection molding	To form samples [31,32,33]
Injection molding	Forming under pressure [34]
	Strain induced [35]
Compression molding	[36]
Melt spinning	[24,37,38]
Electrospinning	[39]
Reactive extrusion	[40,41]
3D printing	[42,43,44]

Note: References list examples of new or varied techniques reported in this review.

**Table 5:** Key enhanced strategies: 2021–2026.

Strategy	Lead	Year	Key Finding
	Author		
Compatibilizer (ESO)	Li [46]	2024	ESO promoted preferential stereocomplex
			crystallization under non-isothermal conditions.
Double-grafted copolymer	Yuan [28]	2024	Only 0.5 wt% additive significantly enhanced
			stereocomplex crystallization and suppressed
			homocrystals.
Regenerated cellulose-assisted	Zhang [21]	2022	Exclusive formation of stereocomplex crystals with
Pickering emulsion			enhanced melt stability and heat resistance.
Graphite oxide addition	Zhong [77]	2023	2 wt% graphite oxide enabled formation of only
			stereocomplex crystallites in
			high-MW PLLA/PDLA blends.
scPLA nucleating agent	Purnama [47]	2021	scPLA nucleating agent improved PLLA properties
			more effectively than PDLA.
Low-MW scPLA powder	Baimark [48]	2022	Increased crystallization rate and enabled complete
nucleation			scPLA crystallites in HMW PLLA.
Self-nucleation by scPLA	Trofin [49]	2025	Increased crystallinity, altered morphology, and
			improved heat deflection temperature.
PVAc-modified cellulose	Ren [78]	2022	Enhanced chain mobility, suppressed d
nanofibers			homocrystallization, promoted scPLA formation.
nanocrystals	Wu [50]	2025	PVAc nanocrystals had a similar effect
Fast solvent evaporation	Liu [19]	2020	Rapid solvent evaporation
(spin coating)			suppressed phase separation and
			enabled exclusive scPLA crystallization.
Thermal control	Yao [20]	2021	Optimized thermal history
during melt crystallization			improved scPLA crystal yield.
Chain alignment	Tuccitto [35]	2022	Chain alignment enhanced scPLA crystallization
Reduced chain entanglement	Sun [60]	2021	Lower chain entanglement increased
			stereocomplex crystal proportion.
Heat-setting of fibers	Sun [60]	2021	Nearly complete stereocomplex structure
			obtained at 190 °C with enhanced
			hydrolysis resistance.
Enhancing intermolecular	Zha [25]	2026	Melt crystallization with Zn(OAc)_2_ at 0.01 wt%
interactions			

## Data Availability

No new data were created or analyzed in this study. Data sharing is not applicable.

## Abbreviations

The following abbreviations are used in this manuscript:

PLA	Polylactic Acid
PLLA	Poly l-lactic Acid
PDLA	Poly d-lactic acid
scPLA	Stereo-complexed polylactic acid
PBS	Poly(butylene succinate)
PMMA	Polymethylmethacrylate
PEG	Polyethylene glycol
PHEEAA	Poly(N-hydroxyethylacetykamide)
PDMAEMA	poly(2-(dimethylamino) ethyl methacrylate)
PCL	Poly(ϵ-caprolactone)
PVAc	Poly(vinyl acetate)
TPS	Thermoplastic starch
CA	Cinnamic acid
HA	Hydroxyapatite
2HB	l-2-hydroxybutanoic acid
Bn	Benzyl alcohol
CD	α-cyclodextrin
ESO	Epoxidized soybean oil
ECD	Branched cardanol ether
PGA	Polyglycolide
PPC	Poly(propylidene carbonate)
GO	Graphene oxide
